# Rapamycin transiently induces mitochondrial remodeling to reprogram energy metabolism in old hearts

**DOI:** 10.18632/aging.100881

**Published:** 2016-02-11

**Authors:** Ying Ann Chiao, Stephen C. Kolwicz, Nathan Basisty, Arni Gagnidze, Julia Zhang, Haiwei Gu, Danijel Djukovic, Richard P. Beyer, Daniel Raftery, Michael MacCoss, Rong Tian, Peter S. Rabinovitch

**Affiliations:** ^1^ Department of Pathology, University of Washington, Seattle, WA 98195, USA; ^2^ Mitochondria and Metabolism Center, Department of Anesthesiology and Pain Medicine, University of Washington, Seattle, WA 98195, USA; ^3^ Department of Environmental Health, University of Washington, Seattle, WA 98195, USA; ^4^ Department of Genome Sciences, University of Washington, Seattle, WA 98195, USA

**Keywords:** cardiac aging, rapamycin, autophagy, mitochondrial metabolism, substrate utilization

## Abstract

Rapamycin, an inhibitor of mTOR signaling, has been shown to reverse diastolic dysfunction in old mice in 10 weeks, highlighting its therapeutic potential for a poorly treatable condition. However, the mechanisms and temporal regulation of its cardiac benefits remain unclear. We show that improved diastolic function in old mice begins at 2-4 weeks, progressing over the course of 10-week treatment. While TORC1-mediated S6 phosphorylation and TORC2 mediated AKT and PKCα phosphorylation are inhibited throughout the course of treatment, rapamycin inhibits ULK phosphorylation and induces autophagy during just the first week of treatment, returning to baseline at two weeks and after. Concordantly, markers of mitochondrial biogenesis increase over the first two weeks of treatment and return to control levels thereafter. This transient induction of autophagy and mitochondrial biogenesis suggests that damaged mitochondria are replaced by newly synthesized ones to rejuvenate mitochondrial homeostasis. This remodeling is shown to rapidly reverse the age-related reduction in fatty acid oxidation to restore a more youthful substrate utilization and energetic profile in old isolated perfused hearts, and modulates the myocardial metabolome *in vivo*. This study demonstrates the differential and dynamic mechanisms following rapamycin treatment and highlights the importance of understanding the temporal regulation of rapamycin effects.

## INTRODUCTION

Aging is the strongest risk factor for cardiovascular disease. The Framingham Heart Study and the Baltimore Longitudinal Study on Aging have shown that even in healthy individuals without concomitant cardiovascular diseases, aging results in a decline in diastolic function, an increase in the prevalence of left ventricular hypertrophy, and relatively preserved systolic function at rest but a decline in exercise capacity and myocardial performance [1]. All of these features are also observed in aging mice [2, 3], suggesting that the phenotypes of cardiac aging are conserved across species. Unlike the well-characterized phenotypes of cardiac aging, the underlying mechanisms of cardiac aging are relatively unclear. Presently, no intervention has been developed to target cardiac aging or to treat diastolic heart failure or heart failure with preserved ejection fraction (HFpEF), which is increasing in prevalence, especially in aged women.

Calorie restriction (CR) is the most-studied longevity intervention and life-long CR has been shown to attenuate age-related impairment in diastolic function in mice [4]. Mechanistic target of rapamycin (mTOR) is one of the major pathways whose inhibition confers the beneficial effects of CR, as shown in multiple aging models [5]. Rapamycin is a well-established inhibitor of mTOR and in a recent study, we showed that both late-life CR and rapamycin treatment for 10 weeks reversed the age-related decline in diastolic function and cardiac hypertrophy in old mice. Rapamycin treatment increases expression of proteins involved in mitochondrial energy metabolism and restores a more youthful proteomic signature in old hearts [6]. Another study also reported a reversal of age-related cardiac dysfunction, accompanied by a suppression of inflammation in hearts of late-life rapamycin treated mice [7]. As the effects of rapamycin in old hearts have hitherto been studied at the treatment endpoint [6, 7], the temporal regulation has not been established. While these studies highlight the potential of rapamycin as an intervention to treat cardiac aging, the mechanisms of its benefits remain unclear. Rapamycin differentially inhibits different mTORC1 substrates in vitro based on the sensitivity of phosphorylation sites [8], but the responses and temporal regulation of different substrates in vivo have not been compared.

The heart is highly metabolically active and continuously consumes ATP for cardiomyocyte contraction/relaxation and regulation of necessary ion pumps. Consequently, impaired myocardial energetics has been reported in multiple models of heart failure (HF) [9]. Mitochondrial dysfunction and altered substrate utilization are components of these energetic changes. Studies have shown reduced fatty acid (FA) substrate utilization in HF but changes in glucose utilization are less consistent [9]. While the experimental evidence is less convincing, the aging heart is believed to display a similar substrate shift as the failing heart [10]. Autophagy (or mitophagy) and mitochondrial biogenesis coordinately regulate mitochondrial homeostasis and are, therefore, critical for normal mitochondrial metabolism and function [11]. Despite the increase in levels of mitochondrial proteins after 10 weeks of rapamycin treatment, no elevation of mitochondrial biogenesis and autophagy markers was observed at this time [6].

We hypothesized that rapamycin treatment induces highly dynamic changes, with differential temporal regulation, to its targets to mediate improvement in cardiac function in old hearts. In this study, we investigated the time course of cardiac remodeling induced by rapamycin to provide insight into the mechanisms of its benefit to cardiac aging. Here, we present evidence that rapamycin induces autophagy and mitochondrial biogenesis transiently to improve mitochondrial metabolism which reaches equilibrium after 10 weeks.

## RESULTS

### Reversal of cardiac aging phenotypes initiates within 2 weeks of rapamycin treatment

**Figure 1:**
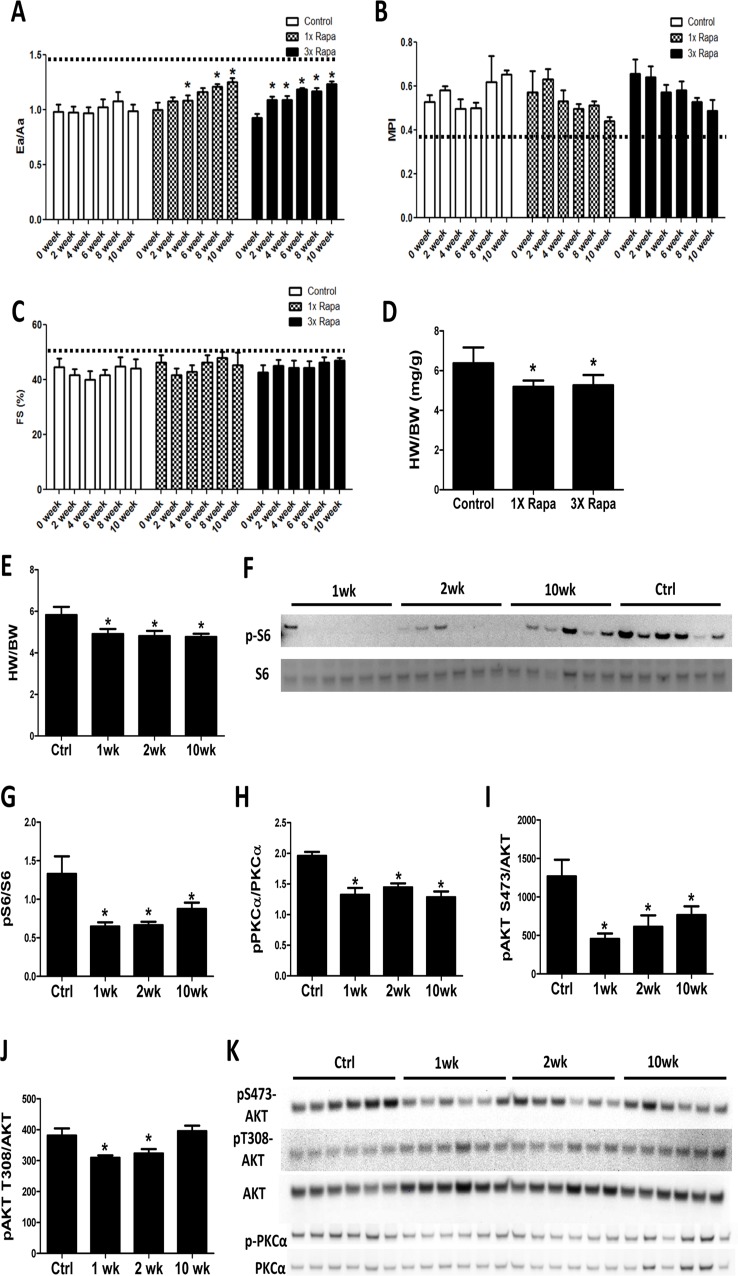
Rapamycin improves cardiac function and inhibits mTOR signaling in old hearts Rapamycin treatment increased Ea/Aa (**A**) and reduced MPI (**B**) but did not alter fractional shortening (FS; **C**). n=3-7/group. * p<0.05 compared to 0 week of corresponding diet. Dotted lines indicate young values. Regression with time was significant (p<0.05) for 1X and 3X Rapa Ea/Aa and 3X MPI. (**D**) 1X Rapa and 3X Rapa treatments showed similar reduction in normalized HW (HW/BW) in old hearts. * p<0.05 compared to old controls. (**E**) 1XRapamycin reversed cardiac hypertrophy at all time points in old hearts. (**F**-**G**) Rapamycin inhibited phosphorylation of S6 ribosomal protein (at S235/S236) at all time points. (**H**-**K**) Rapamycin reduced phosphorylation of PKCα (**H**) and S473-AKT (**I**) at all time points and T308-AKT (**J**) at 1- and 2-week. * p<0.05 compared to old controls (Ctrl). Data are represented as mean ± SEM.

To determine the time course of cardiac remodeling by rapamycin treatment, echocardiography was performed every other week on mice receiving control or rapamycin diets for a 10-week period. We observed that mice treated with 2.24 mg/kg/day rapamycin (1X Rapa, the initially described NIA Interventions Testing Program [ITP] dose) and 6.72 mg/kg/day rapamycin (3X Rapa) both showed a similar progressive improvement in the diastolic function parameter Ea/Aa (Fig. 1A) over 10 weeks of treatment (p<0.05 for both trend analyses). This improvement in diastolic function was detectable as early as 2-4 weeks in both doses of rapamycin. At the same time, 1X and 3X rapamycin treated mice also showed reduced myocardial performance index (MPI) over time (Fig. 1B) (p<0.05 for both trends). Systolic function was relatively preserved with age and did not change with rapamycin treatment (Fig. 1C). We measured heart weight normalized to body weight (HW/BW) and observed lower HW/BW in 1XRapa- and 3X Rapa-treated mice compared to controls (p<0.05 for both; Fig 1D). The lower normalized HW agrees with our previous result in 1X Rapa mice [6] and indicates that these doses of rapamycin can regress age-dependent cardiac hypertrophy. The observation that 1X and 3X Rapa showed similar cardiac function and hypertrophy improvement suggests that 1X (2.24 mg/kg/day) rapamycin is sufficient for reversal of cardiac aging phenotypes in these female mice. To delineate the mechanisms of this benefit, we fed a separate cohort of mice with diet containing 2.24 mg/kg/day rapamycin for 1, 2 and 10 weeks to investigate the acute and chronic molecular changes induced by rapamycin treatment. With this latter cohort, we found that reduced normalized heart weight appeared as early as 1-week following treatment, and persisted thereafter (Fig. 1E).

### Rapamycin inhibits canonical mTOR targets throughout the course of treatment

A decade before its beneficial effects on aging hearts was demonstrated, rapamycin was known to prevent and even regress pressure-overload induced cardiac hypertrophy, presumed to be mediated partly via inhibition of the TORC1 target S6 kinase [12-14]. We studied this in the aging heart by assessing phosphorylation of the S6 kinase downstream target, ribosomal protein S6. Rapamycin greatly reduced phosphorylation of S6 in old hearts at 1-, 2-, and 10- week time points (Fig. 1F-G). While it was originally thought that the TORC2 pathway was rapamycin-insensitive, recent studies have shown that rapamycin inhibits TORC2 signaling subacutely [15, 16]. We found reduced phosphorylation of TORC2 targets, AKT (at S473) and PKCα (at S657), in hearts at all times studied (1-, 2-, and 10-week; Fig. 1H-I and K). Phosphorylation of AKT at T308, a PDK1 site, was slightly reduced at 1 and 2 weeks after treatment (Fig 1J-K), that may be due to a small increase in total AKT levels. These results indicate that rapamycin inhibits TORC1 and TORC2 signaling throughout the course of treatment.

### Rapamycin inhibits ULK to induce autophagy transiently at 1 week of treatment

**Figure 2:**
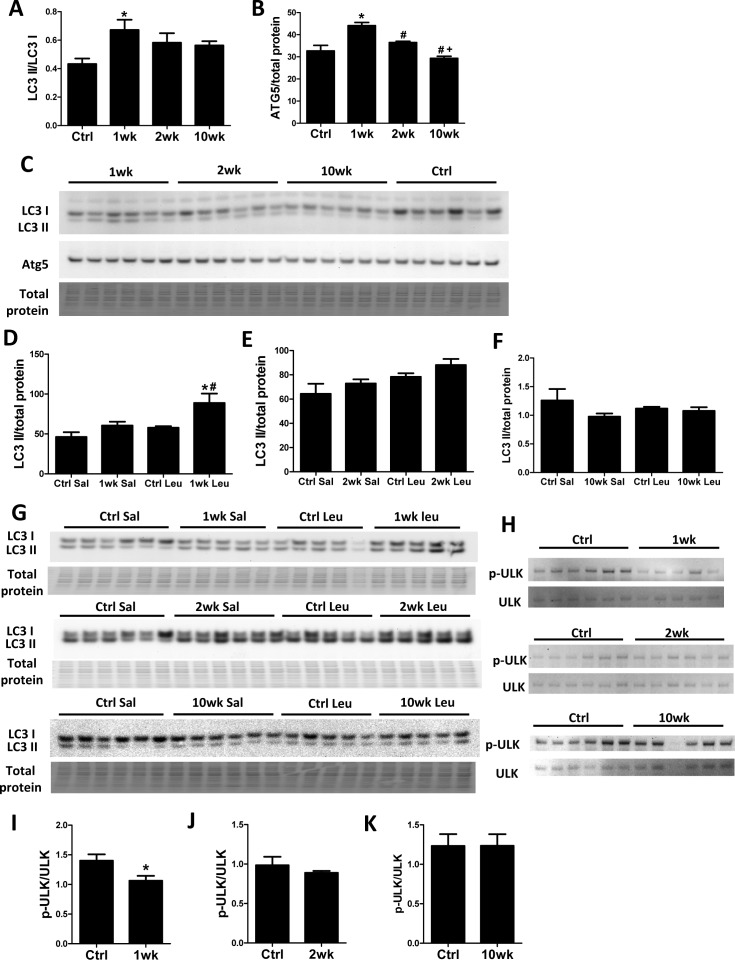
Autophagy increases transiently at 1 week after rapamycin treatment initiation Rapamycin increased levels of autophagic markers LC3 II/I (**A**) and ATG5 (**B**) in old hearts. * p <0.05 vs. old Ctrl; # p <0.05 vs. 1wk; + p<0.05 vs. 2 wk. (**C**) Immunoblotting showed increased LC3 II/LC3 I ratio and Atg5 levels after 1-week rapamycin treatment which returned towards the control level after 2 weeks. (**D**-**G**) Rapamycin induced accumulation of LC3 II after leupeptin injection at 1 week (**D, G**) but not 2 weeks (**E**, **G**) or 10 weeks (**F**, **G**) after treatment initiation. * p <0.05 vs. 1wk Saline; # p<0.05 vs. Ctrl Leupeptin. Immunoblotting of ULK1 (**H**) showed that rapamycin inhibited ULK phosphorylation at S757 at 1-week (**I**) but not 2-week (**J**) and 10-week time points (**K**). n≥5/group. * p <0.05 vs. Ctrl. Sal, saline; Leu, leupeptin. Data are represented as mean ± SEM.

Recent studies have shown that TORC1 inhibits autophagy by direct phosphorylation of ULK1 [17, 18]. We previously showed that 10-week rapamycin treatment does not alter levels of autophagy markers, including LC3 II/I, in old hearts [6]. Consistent with our previous study, the LC3 II/LC3 I ratio did not change after 10 weeks of rapamycin treatment. However, we saw an increased LC3 II/LC3 I ratio after 1-week rapamycin treatment which returned towards the control level after 2 weeks (Fig. 2A and C). Similarly, levels of ATG5 increased at 1-week but not at later time points (Fig 2B-C). To verify that increased levels of these autophagy markers were reflective of an increase in autophagic flux, we measured the accumulation of LC3 II after intraperitoneal injection of protease inhibitor leupeptin [19]. We observed that autophagic flux was indeed increased in hearts at 1 week but not at 2 weeks or 10 weeks after initiation of treatment (Fig. 2D-G). While canonical TORC1 signaling was inhibited throughout the course of rapamycin treatment, ULK phosphorylation (at S757) was only inhibited at 1 week and returned to near the control levels at 2 weeks and 10 weeks, showing similar kinetics as the autophagic flux (Fig. 2H-K).

### Rapamycin induces mitochondrial biogenesis during the first 2 weeks of treatment

**Figure 3:**
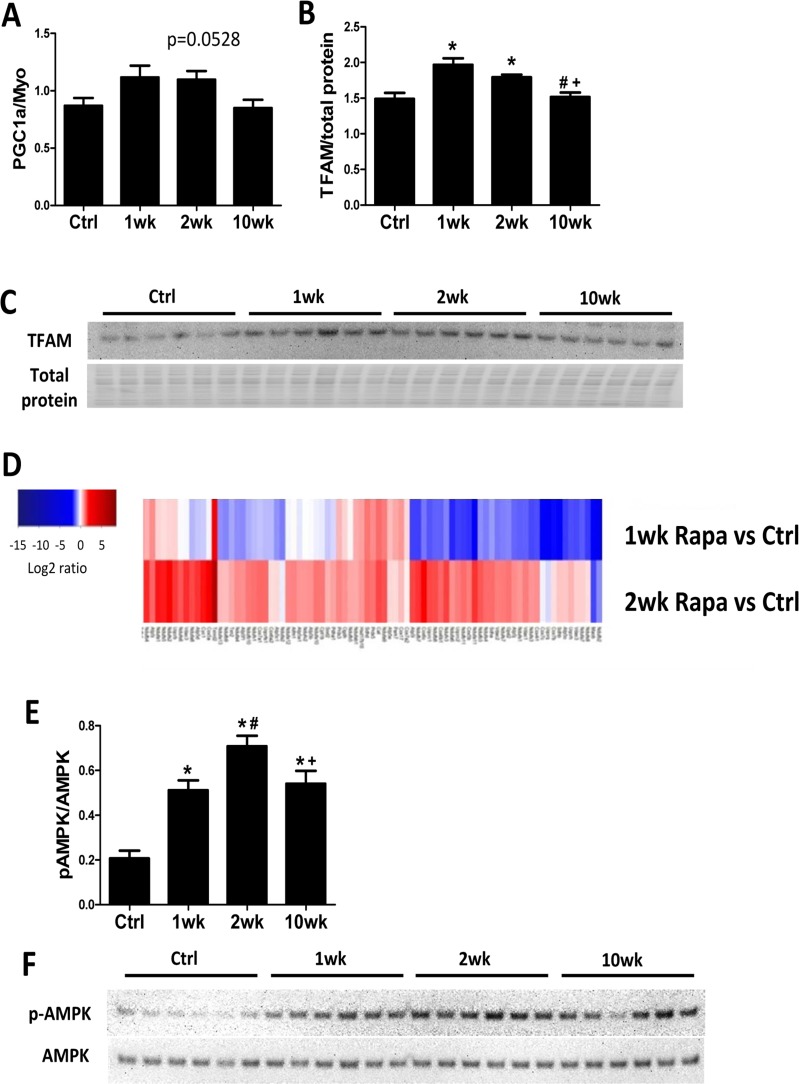
Mitochondrial biogenesis increases in the first 2 weeks of rapamycin treatment Rapamycin transiently induced expression of PGC-1α (**A**) and TFAM (**B**-**C**) at the first two week of treatment. (**D**) Heatmap for abundance of proteins in mitochondrial function pathway at 1 and 2 weeks compared to controls, with red indicates higher and blue indicates lower abundance. (**E**-**F**) Rapamycin induced AMPK phosphorylation at all time points. n≥5/group. * p <0.05 vs. old Ctrl; # p <0.05 vs. 1wk; + p<0.05 vs. 2 wk. Data are represented as mean ± SEM.

We previously showed that 10-week rapamycin treatment reversed the age-related decrease in levels of mitochondrial proteins without increasing mitochondrial biogenesis [6]. We assessed mitochondrial biogenesis at different time points to determine if there was a transient increase in mitochondrial biogenesis to confer the proteomic changes. By real-time PCR, expression of the mitochondrial biogenesis marker PCG-1α increased in the first 2 weeks of rapamycin treatment but returned to control levels at 10 weeks (Fig 3A). Protein expression of its downstream transcriptional factor, mitochondrial transcriptional factor A (TFAM), also increased by 1- and 2-week but not 10-week rapamycin treatment (Fig. 3B-C). The increase in autophagy and mitochondrial biogenesis in the first two weeks of treatment suggests that old mitochondrial components can be removed and replaced my newly synthesized mitochondria during this time. Concordantly, MS based global proteomic analysis showed a mixture of increased and reduced levels of mitochondrial proteins after 1-week treatment, when autophagy and mitochondrial biogenesis both increased, and a general increase in levels of mitochondrial proteins after 2-week treatment, when autophagy returned to normal and mitochondrial biogenesis increased (Fig. 3D). The proteomic changes support the hypothesis that rapamycin induces mitochondrial remodeling within 2 weeks of initiating treatment to replenish mitochondrial proteome and improve mitochondrial metabolism in old hearts.

Adenosine monophosphate–activated protein kinase (AMPK) promotes mitochondrial biogenesis and regulates cellular energy metabolism [20]. Recently, rapamycin treatment was shown to induce AMPK phosphorylation (activation) in cultured cells [21] but its action *in vivo* has not been established. We found that rapamycin increased AMPK phosphorylation at all time points (Fig 3E-F), with peak levels at 2-week.

### Rapamycin reverses age-related reduction in fatty acid oxidation (FAO) in old heart and enhances mitochondrial metabolism

**Figure 4:**
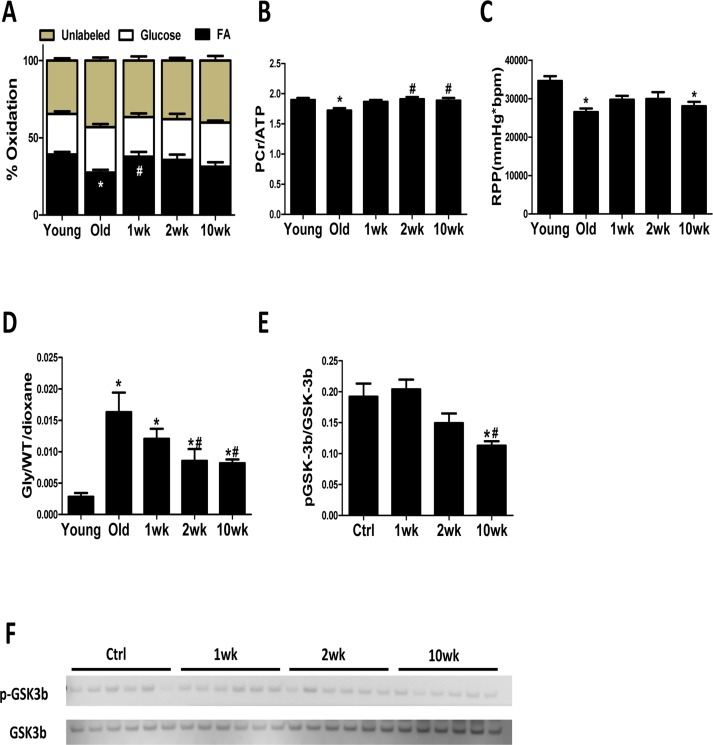
Cardiac energy metabolism is rejuvenated after rapamycin treatment (**A**) Contribution of FAO to energy metabolism was reduced in old control hearts, but not old hearts treated with rapamycin, compared to young hearts. (**B**) ^31^P NMR spectroscopy showed reduced PCr/ATP ratio in old compared to young control heart which was reversed by 2-week and 10-week of treatment. (**C**) Old hearts showed reduced cardiac function, represented by lowered rate pressure product (RPP), when compared to young controls. (**D**) Old hearts displayed a large increase in the incorporation of ^13^C glucose into glycogen during perfusion that was attenuated in rapamycin treated hearts. * p <0.05 vs. Young; # p <0.05 vs. Old. (**E**-**F**) Rapamycin treatment reduced GSK-3β phosphorylation. n≥6/group. * p <0.05 vs. old Ctrl; # p <0.05 vs. 1wk. Data are represented as mean ± SEM.

To determine if the transient induction in autophagy and mitochondrial biogenesis leads to improved mitochondrial metabolism in the old heart, we measured substrate utilization in isolated perfused hearts from control and rapamycin-treated mice. FAO was reduced by 30% in old control hearts compared to young controls, and this was accompanied by decreased PCr/ATP ratio and reduced cardiac function ( (Fig. 4A-C). Strikingly, 1 week of rapamycin treatment reversed this age-related reduction in FAO (Fig. 4A). Although 2 and 10 weeks of rapamycin treatment did not significantly increase FAO above old hearts, the PCr/ATP ratio was normalized at these later time points (Fig. 4A-B). LV developed pressure, heart rate and coronary flow were not different between groups (Fig. S1A-C). Overall, these findings suggest that rapamycin treatment leads to an enhanced utilization of substrates that results in the recovery of impaired myocardial energetics in old hearts.

We also observed a dramatic increase in the incorporation of ^13^C glucose into glycogen in old mouse hearts, suggesting an increased storage of exogenously supplied glucose (Fig. S1D). This age-effect was largely attenuated by rapamycin treatment at 2 to 10 weeks (Fig. 4D) and coincided with reduced phosphorylation (i.e. derepression) of glycogen synthase kinase (GSK)-3β, a downstream target of AKT [22] that inhibits glycogen synthase (Fig. 4E).

### Cardiac energy metabolism is rapidly remodeled by rapamycin treatment

**Figure 5:**
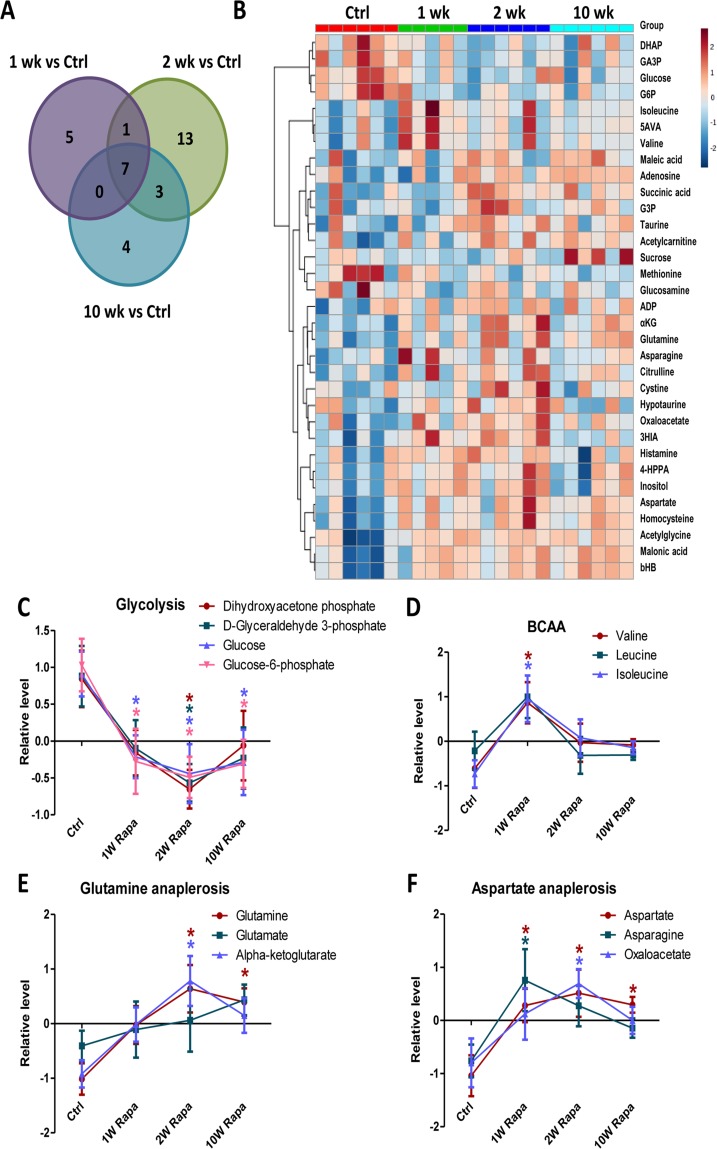
Rapamycin induces differential temporal regulation on cardiac metabolome Venn diagram (**A**) and heatmap (**B**) of metabolite changes for different durations of rapamycin treatment. Rapamycin differentially regulated levels of metabolites in glycolysis (**C**), BCAA (**D**), glutamine anaplerosis (**E**) and aspartate anaplerosis (**F**). n≥5/group. * p <0.05 vs. Ctrl. Data are represented as mean ± SEM.

We performed metabolic profiling of heart extracts to characterize the *in vivo* metabolic phenotypes of hearts from rapamycin treated mice. We measured levels of 130 metabolites in the heart samples. Of these, 33 had significantly different levels in at least one of the treatment time points compared to control hearts (p<0.05, Fig. 5A and Table 1). Fig 5B shows a heatmap of the 33 metabolites ordered by clustering of the pattern across the 4 groups. From this, we identified metabolic pathways that displayed differential temporal regulation by rapamycin treatment. In particular, the levels of 4 glycolytic intermediates decreased at 1-, 2- and 10-week, consistent with reduced glycolysis after rapamycin treatment (Fig 5C). We previously demonstrated that 10-week rapamycin treatment reduced glycolysis, and we now see that the reduced glycolysis is initiated acutely at 1 week. We also found that the levels of branched-chain amino acids increased (for valine and isoleucine) or trended towards an increase (for leucine) at 1-week but returned to control levels at later time points (Fig 5D). Levels of α-ketoglutarate and oxaloacetate, two TCA cycle intermediates that are entry points of anaplerosis, were increased, as were levels of glutamine, aspartic acid and asparagine, all substrates for anaplerosis, beginning within 2 weeks of rapamycin treatment (Fig 5E-F). These findings suggest that the rapamycin induced increase in TCA cycle intermediates may be a result of increased anaplerosis.

**Table 1:** Metabolites that were significantly different in at least one of the rapamycin treatment time points compared to control. Fold changes with p-value < 0.05 are bold and italic

Metabolites	1wk vs Ctrl		2wk vs Ctrl		10wk vs Ctrl	
	Fold change	p-value	Fold change	p-value	Fold change	p-value
Glucose	***0.58***	0.030	***0.54***	0.012	***0.58***	0.023
Glucose 6-phosphate	***0.33***	0.010	***0.29***	0.003	***0.33***	0.008
Aspartate	***1.6***	0.020	***1.8***	0.003	***1.7***	0.010
Homocysteine	***1.7***	0.007	***1.9***	0.001	***1.7***	0.004
Acetylglycine	***2.3***	0.005	***2.4***	0.002	***2.4***	0.002
Malonic acid	***1.9***	0.012	***2.2***	0.002	***2.3***	0.001
β-Hydroxybutyric acid	***1.9***	0.011	***2.2***	0.002	***2.3***	0.001
3-Hydroxyisovaleric acid	***1.3***	0.023	***1.4***	0.002	1.1	0.278
Glyceraldehyde 3-phosphate	0.48	0.059	***0.36***	0.008	***0.45***	0.034
Glutamine	1.1	0.190	***1.2***	0.004	***1.2***	0.012
Acetylcarnitine	1.1	0.335	***1.4***	0.009	***1.3***	0.023
Isoleucine	***1.3***	0.017	1.2	0.148	1.1	0.295
5-Aminovaleric acid	***1.3***	0.041	1.1	0.291	1.1	0.368
Valine	***1.3***	0.045	1.1	0.318	1.1	0.374
Glucosamine	***0.57***	0.049	0.79	0.384	0.72	0.218
Asparagine	***1.4***	0.022	1.3	0.063	1.2	0.258
Dihydroxyacetone phosphate	0.41	0.054	***0.27***	0.006	0.46	0.077
Succinic acid	0.96	0.896	***2.1***	0.009	1.6	0.090
Glyceraldehyde 3-phosphate	0.85	0.452	***1.5***	0.046	1.2	0.403
Taurine	1.1	0.362	***1.3***	0.021	1.2	0.139
ADP	1.4	0.294	***2.1***	0.031	1.6	0.144
α-Ketoglutarate	1.1	0.245	***1.3***	0.005	1.2	0.065
Citrulline	1.2	0.090	***1.4***	0.012	1.2	0.120
Cystine	1.0	0.981	***1.5***	0.025	1.1	0.705
Hypotaurine	1.2	0.397	***1.6***	0.033	1.0	0.868
Oxaloacetate	1.2	0.189	***1.3***	0.010	1.2	0.146
Histamine	1.6	0.065	***1.8***	0.018	1.1	0.634
4-Hydroxyphenylpyruvic acid	1.3	0.201	***1.4***	0.049	1.1	0.626
Inositol	1.3	0.094	***1.4***	0.029	1.1	0.643
Maleic acid	1.1	0.533	1.2	0.243	***1.3***	0.048
Methionine	0.77	0.154	0.74	0.091	***0.66***	0.026
Adenosine	1.1	0.762	1.9	0.059	***2.5***	0.011
Sucrose	0.74	0.437	0.89	0.754	***2.2***	0.047

## DISCUSSION

### Induction of autophagy by rapamycin is transient

TORC1 has been shown to inhibit autophagy by direct phosphorylation of ULK1 [17, 18]. Nevertheless, no elevation of autophagy markers was observed in old hearts after 10 weeks of rapamycin treatment [6]. Here, we show that rapamycin induces autophagy rapidly (at 1 week after treatment initiation) by relieving inhibitory S757 phosphorylation of ULK1. Interestingly, the suppression of S757 phosphorylation of ULK1 and autophagic flux return to and stay at control levels from 2 weeks to 10 weeks of treatment. This is the first time this transient induction of autophagy by rapamycin has been shown; however, it makes sense in the context of reduced nutrient signaling, as sustained autophagy could not be easily supported in conditions of energy and substrate limitation, as would be seen in CR.

### Rapamycin improves mitochondrial energy metabolism in old hearts

The heart has a high energy demand to sustain its contractile function and can use both glucose and FA as fuel to generate energy. However, FA are the preferred substrates under normal conditions, as their mitochondrial oxidation has the highest energetic yield [23]. A shift in substrate utilization from FA to glucose is observed in HF and is implicated in its pathogenesis [24]. A similar metabolic shift away from FA utilization has also been demonstrated in the aging heart [25, 26]. Mitochondrial function and expression of mitochondrial enzymes in FAO, TCA cycle and the respiratory chain proteins are critical determinants of substrate metabolism. We previously demonstrated that 10-week rapamycin treatment increases expression of these proteins and reduces expression of glycolytic enzymes, suggesting a potential reversal of substrate shift [6]. Using isolated heart perfusion with ^13^C-labeled substrates, we provide direct evidence that rapamycin increases FAO in old hearts after only 1 week of treatment. This is also associated with the normalization of myocardial bioenergetics (PCr/ATP) in the old hearts. Activation of mTOR signaling stimulates glucose uptake and glycolysis, and rapamycin suppresses these processes [27, 28]. Previous study has shown that rapamycin increases FAO and reduces glucose utilization through inhibition of mTOR signaling in skeletal muscle cells [29]. Similarly, we now show that rapamycin increases FAO in old hearts and reverses the age-related substrate shift, and that this shift is a probable component of the improved cardiac function.

AMPK is activated by nutrient or energy deprivation and is a well-characterized upstream regulator of TORC. Habib et al recently showed that rapamycin treatment induces AMPK phosphorylation (activation) in cultured cells [21]. The effect of rapamycin on AMPK activity *in vivo*, however, has not been established. We showed that rapamycin increased AMPK phosphorylation in old hearts throughout the course of treatment. AMPK promotes mitochondrial biogenesis and regulates cellular energy metabolism [20]. Thus, rapamycin induced AMPK activation may contribute to the enhanced mitochondrial biogenesis and improved mitochondrial metabolism in old hearts.

### Rapamycin induces metabolic remodeling and alters myocardial metabolome

Excess glycogen accumulation can lead to cardiomyopathy in glycogen storage diseases (GSD) [30-32]. A recent study showed that rapamycin can reduce glycogen accumulation in muscle cells from GSD IIIa patients and GSD IIIa dogs at advanced age [33]. We observed a drastic age-related increase in incorporation of glucose into glycogen in the heart, and that rapamycin greatly suppresses the incorporation of glucose into glycogen in old hearts. The suppression by rapamycin is associated with derepression of GSK-3β, a negative regulator of glycogen synthase. GSK-3β is a downstream target of AKT [22], the inhibition of AKT by rapamycin may cause the reduced phosphorylation of GSK-3β. The precise role of glycogen regulation in cardiac aging and the effects of rapamycin require further investigation.

By metabolomic analysis, we demonstrated that rapamycin treatment altered the levels of metabolites in multiple metabolic pathways, including glycolysis, branched chain amino acid and anaplerosis. These changes in metabolite levels displayed different time-course but mostly initiated at 1 or 2 weeks of rapamycin treatment. Overall, we showed that rapamycin treatment modulated myocardial metabolome in old hearts and that most of these changes began within 2 weeks after initiation of treatment.

### Differential temporal regulation of downstream targets by rapamycin

Rapamycin is a widely used TORC1 inhibitor, but some TORC1 functions have been shown to be resistant to rapamycin [34]. Kang and colleagues recently showed that rapamycin differentially inhibits different mTORC1 substrates *in vitro* based on the sensitivity of phosphorylation sites [8]. This study demonstrated that the temporal regulation of different TORC substrates *in vivo* is another critical factor to consider when using rapamycin.

Rapamycin binds and allosterically inhibits TORC1 but also inhibits TORC2 signaling by disruption of complex assembly [15, 16]. We showed that rapamycin inhibits canonical TORC1 and TORC2 signaling (S6, AKT and PKCα phosphorylation) throughout the 10-week course of treatment. On the other hand, rapamycin only transiently suppresses TORC1-mediated ULK phosphorylation and induces autophagy during the first week of treatment. The induction of mitochondrial biogenesis by rapamycin have a different time-course and stay elevated in the first 2 weeks of treatment before returning to control levels. In addition, metabolomic analysis also revealed that rapamycin modulates different metabolic pathways with different time-courses. This evidence suggests that rapamycin exerts differential temporal regulation on its downstream targets and highlights the importance of understanding the temporal regulation of an intervention such as rapamycin. As long-term rapamycin treatment is associated with a number of significant side effects, our observations provides novel insight into the design of improved strategies to safely implement this intervention to improve health and physiologic function, including cardiac performance, in the elderly population.

In conclusion, we showed that rapamycin inhibits canonical TORC1 and TORC2 downstream targets in old hearts throughout the course of 10 week treatment. However, its effects on induction of autophagy and mitochondrial biogenesis are more transient and take place within 2 weeks of treatment. This acute remodeling reverses the age-related reduction in FA utilization and is associated with improved cardiac energy metabolism. These benefits persist throughout the course of treatment, and seem likely to mediate the observed improvements in cardiac function. As there is no present treatment for age-related diastolic failure, the results of this study provide novel insight for the design and implementation of new effective therapies.

## MATERIALS AND METHODS

### Animals

Young (3-5 month-old) and old (24-26 month-old) C57BL/6 female mice were obtained from the National Institute of Aging Charles River colony. Females were used, as the effects of rapamycin in murine aging are much larger in this gender [35]. Mice were housed at 20°C in an AAALAC accredited facility under Institutional Animal Care and Use Committee (IACUC) supervision.

For the longitudinal study, old mice were randomly assigned to three groups and fed with: control diet, diet with microencapsulated rapamycin at 2.24 mg/kg/day (1X Rapa, 14 ppm rapamycin in diet, Rapamycin Holdings, San Antonio TX) or 6.72 mg/kg/day (3X Rapa, 42 ppm rapamycin in diet), and maintained on the corresponding diets for up to 10 weeks. Echo-cardiography was performed under 0.5-1% isoflurane at baseline and every other week until the endpoint using a Siemens Acuson CV-70 equipped with a 13MHz probe. At endpoint, mice were euthanized by cervical dislocation for tissue harvest.

For the time-course study, old mice were assigned to four groups and were fed with: 1) control diet; 2) 1X Rapa-containing diet for 10 weeks (10wk); 3) control diet then switched to 1X Rapa-containing diet 2 weeks before endpoint (2wk); and 4) control diet then switched to 1X Rapa-containing diet 1 week before endpoint (1wk). At 10 weeks, hearts were harvested for biochemical assays or isolated heart perfusion. For autophagic flux assay, mice were injected intraperitoneally with 40mg/kg leupeptin hemisulfate (Fisher Scientific) or saline vehicle 1 hour before tissue harvest. Mice were euthanized by cervical dislocation for tissue harvest.

### Real-time PCR

Total RNA was isolated with TRIzol reagent and RNeasy kit (Qiagen). cDNA was synthesized and quantitative real-time PCR was performed using ABI gene expression assay for PGC-1α with Rotor-Gene Q.

### Immunoblotting

Proteins were extracted from heart tissues with K150T buffer (150 mM KCl, 50 mM Tris-HCl pH7.4, 0.125% Na deoxycholate, 0.375%Triton X-100, 0.15% NP-40, 4mM EDTA, 50 mM NaF) and immunoblotting was performed as previously described [3]. In brief, equal amount of proteins (15 ug) were resolved on 4-12% NuPAGE Bis-Tris gel and transferred to PVDF membrane. Pierce Reversible Protein Stain Kit was used to detect total proteins for loading normalization. Primary antibodies used in immunoblotting were phospho-S6(Ser235/236), S6, phospho-AKT (S473), phospho-AKT (T308), phospho-ULK (S757), ULK, phospho-AMPK (T172), AMPK, phospho-GSK-3β (S9), GSK-3β, Atg5 (all from Cell Signaling), phospho S657-PKCα, PKCα (both from Santa Cruz), LC3 (from Novus Biologicals), TFAM (mtTFA; from Abcam). Secondary antibodies used were donkey anti-rabbit IgG secondary antibody and goat anti-mouse IgG secondary antibody (both from Thermo Scientific. AlphaView Software was used for image acquisition and signal quantification.

### Mass spectrometry

Pulverized heart tissues were homogenized in ice-cold extraction buffer (250 mM sucrose, 1 mM EGTA, 10 mM HEPES, 10 mM Tris-HCl pH7.4). Lysates were centrifuged at 800 × g for 10 minutes to remove debris. Samples were trypsin digested and purified by MCX column (Waters). LC-MS/MS analysis was performed with a Waters nanoAcquity UPLC and a Thermo Scientific LTQ Orbitrap Velos. The raw data from MS/MS are available at https://chorusproject.org/pages/blog.html#/963. In order to access the data, a free account must be obtained by following the instructions on the Chorus Project website.

Protein abundance was analyzed as previously described [6]. We performed Topograph chromatogram alignment to account chromatographic drift that may occur during the LC-MS/MS and allow comparisons of low abundance analytes that may be detected in some but not all samples [6, 36]. Chromatographic MS1 peak areas were used to quantify abundance.

### Isolated heart perfusions and NMR spectroscopy

Isolated mouse hearts were perfused in Langendorff-mode combined with ^31^P nuclear magnetic resonance (NMR) spectroscopy as previously described [37]. To assess substrate utilization, the perfusate consisted of a modified Krebs-Henseleit buffer supplemented with 0.4mM uniformly labeled ^13^C long-chain fatty acids, 5.5 mM 1-^13^C glucose, 1.2 mM lactate, and 50μU/ml insulin. At the end of the 40 minute perfusion period, hearts were freeze-clamped. The frozen tissue was pulverized under liquid nitrogen, extracted with perchloric acid, neutralized, and lyophilized. The sample was resuspended in deuterium oxide and analyzed via ^13^C NMR spectroscopy. The contributions of glucose and fatty acids was determined by isotopomer analysis of the C3 and C4 glutamate multiplets from the NMR spectra. ^13^C glucose incorporation into glycogen was assessed in isolated perfused hearts combined with dynamic ^13^C NMR spectroscopy [38] or via the peak area normalized to tissue weight obtained via 13C NMR spectroscopy of cardiac tissue extracts.

### Metabolic profiling of cardiac tissue extract

Frozen heart tissues were pulverized and were homogenized in 80:20 methanol:water. Soluble extracts were collected and dried by speed vac. Dried extracts were resuspended in 5 mM ammonium acetate in 95% water/5% acetonitrile + 0.5% acetic acid and filtered. Metabolic profiling by LC-MS was performed as previously described [6].

### Data analysis for metabolic profiling

The relative peak intensity of each metabolite (raw peak intensity/mg of tissue extracted) was the starting point of the statistical analysis of metabolite abundance for Control, 1-week, 2-week, and 10-week rapamycin treatment. The data from 23 samples (n=5-6/group) was analyzed by R/Bioconductor [39] using the limma package [40]. The data were median normalized and log2 converted. The limma methodology calculates a p-value for each metabolite using a modified t-test in conjunction with an empirical Bayes method to moderate the standard errors of the estimated log-fold changes. For the three contrasts of interest, the 33 metabolites with an unadjusted p < 0.05 are shown in Figure 5A and Table 1. The heatmap of the relative peak intensities of the 33 metabolites was generated using Metaboanalyst 3.0 [41] using the same normalization approach. The normalized intensities were autoscaled (ie. the mean intensity of all samples were centered at zero for each metabolites) for visualization of rapamycin effect in different metabolites in Figure 5C-F.

### Statistical analyses

Echocardiographic results were analyzed by paired T-test with baseline and linear regression over time. HW/BW, isolated heart perfusion, real-time PCR and immunoblotting results were analyzed by one-way ANOVA and substrate utilization results were analyzed by two-way ANOVA. Graphpad Prism 5 was used for statistical analyses and all data were plotted as mean ±SEM.

## SUPPLEMENTARY FIGURE


