# Supplementary material for: Rapamycin transiently induces mitochondrial remodeling to reprogram energy metabolism in old hearts
Source: Aging (Albany NY). 2016 Feb 11;8(2):314–26. doi: 10.18632/aging.100881 (PMC4789585; doi:10.18632/aging.100881)
Supplement: Supplementary file 1 [file aging-08-314-s001.pdf]

SUPPLEMENTAL FIGURE

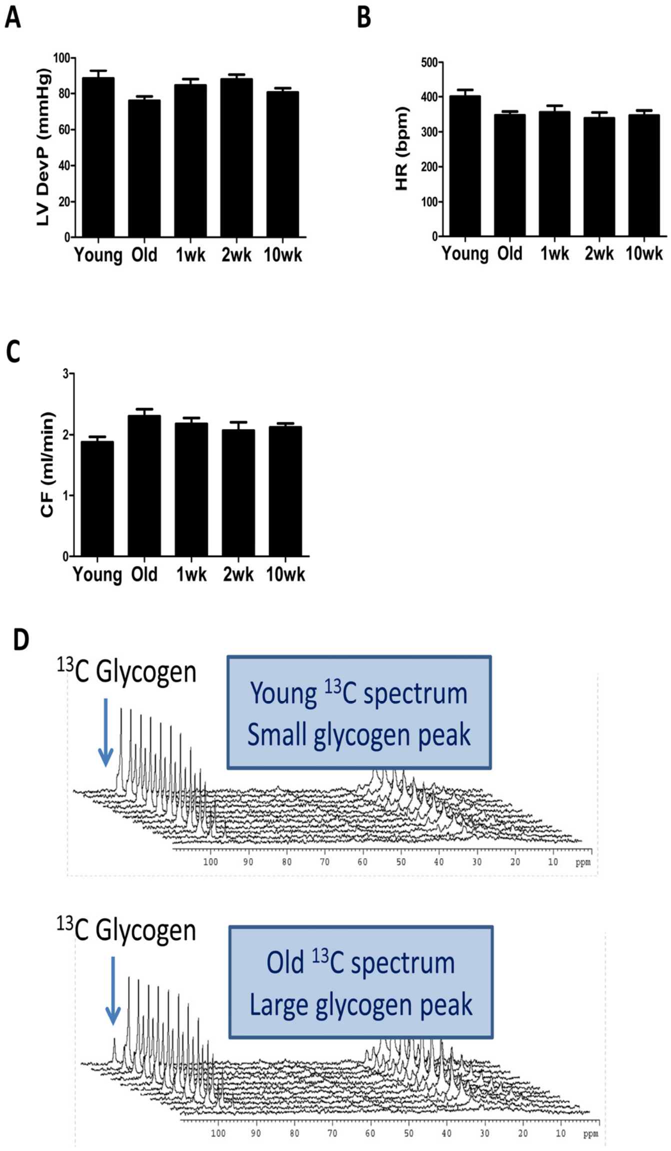

Figure S1. Incorporation of ^{13}C glucose into glycogen increases in old hearts. In isolated perfused heart, LV developed pressure (**A**), heart rate (**B**) and coronary flow (**C**) were not significantly different with aging or rapamycin treatment. (D) Dynamic ^{13}C NMR spectroscopy of isolated perfusion hearts showed a larger ^{13}C glycogen peak, indicating an increase in the incorporation of ^{13}C glucose into glycogen, in old hearts compared to young controls.
